# Contemporary Fire Regimes of the Subtropical Everglades

**DOI:** 10.1038/s41597-025-05917-6

**Published:** 2025-10-07

**Authors:** Sparkle L. Malone, Grace McLeod, Angel Chen, Mayavati Tupaj

**Affiliations:** 1 Yale School of the Environment, Yale University, New Haven, CT 06511 USA; 2 Florida International University, Department of Biological Sciences, Miami, FL 33199 USA; 3 Everglades National Park, Homestead, FL 33034 USA

**Keywords:** Natural hazards, Fire ecology

## Abstract

Fire is a fundamental force that shapes ecosystems by influencing vegetation composition, succession, and structural diversity. Fire regimes, defined by fire frequency, intensity, and seasonality, vary across ecosystems and are critical in fire-dependent landscapes. In the Florida Everglades, fire is a key driver of ecological dynamics, interacting with hydrology and the structure of vegetation. This study defines contemporary fire regimes by describing fire patterns from 1978 to 2023, utilizing fire perimeter data from Everglades National Park and Big Cypress National Preserve. Our findings reveal a highly variable annual burned area with a strong increasing trend. Prescribed fires were the foundation of trends in fire activity, as wildfires remained stable over the study period. Across the Everglades, fire return intervals differed between ecosystems, with upland ecosystems experiencing more frequent fires than wetland ecosystems. Our findings highlight the role of fire management in shaping modern fire regimes and underscore the importance of prescribed burns in maintaining ecosystem function and resilience in the Everglades.

## Background & Summary

Fire shapes ecosystems across Earth’s biomes^1,2^. With varying spatial and temporal patterns that interact to characterize the fire regime, fire influences the biological and structural composition of ecosystems. Fire creates opportunities and space for new individuals to establish themselves, thereby influencing competition, succession, and environmental heterogeneity^3–5^. In ecosystems that experience regular fires, the flora and fauna can evolve to thrive in the presence of fire. Fire-adapted species depend on fire for specific stages of their life cycle, such as seed dispersal or the regeneration of certain species, or for the overall health and structure of the ecosystem. Regular fire is critical in fire-dependent landscapes to maintain biological and structural diversity, support ecosystem function, and maintain resilience^5–9^.

Fire regimes are defined by the frequency, intensity, and seasonality of fires^10,11^. These regimes play a crucial role in shaping ecosystems, as various plants and animals are adapted to particular fire patterns. Fire regimes vary among ecosystems due to differences in climatic conditions, as well as the availability and characteristics of fuels^2^. Fire-dependent ecosystems, such as prairies, savannas, and pine forests, rely on distinct fire regimes to maintain biodiversity and prevent the encroachment of invasive species^12^. However, due to changes in land management and the effects of climate change, fire regimes are shifting across ecosystems. This results in contemporary fire regimes that differ significantly from historical patterns, which can lead to unexpected impacts on ecosystem functions^12–15^.

In the Southeastern United States and the Caribbean Basin, fire regimes are characterized by frequent, low-intensity surface fires, which primarily consume understory and mid-story vegetation^16–19^. In these fire-dependent ecosystems, shifts in fire frequency and intensity can significantly impact the characteristics and successional trajectories of regenerating plant communities^19–21^. Recognizing the importance of fire for adaptive land management, Everglades National Park became the first to establish a fire management program in 1948 and incorporated prescribed burning in the 1950s^22,23^.

Land managers intentionally set prescribed fires to mimic natural fire cycles^24^, which is essential for maintaining the biodiversity and functionality of the Everglades’ unique landscape^25–27^. Regular fires prevent the overgrowth of certain plants, like invasive species and dense vegetation, that would otherwise dominate and alter the landscape^28,29^. Fires also help release nutrients back into the soil, providing a nutrient boost that benefits the growth of plants adapted to this cycle^30^. This is especially important in the nutrient-poor soils of the Everglades^31^. Fires create a mosaic of habitats, ranging from open grasslands to dense forests, which support diverse wildlife populations^32–34^. Many species rely on these varied habitats for feeding, nesting, and shelter^35,36^. Prescribed fires can also reduce the risk of high-severity wildfires^37,38^, which can cause more extensive damage to the ecosystem, nearby communities, and endangered species. Fires also help clear out dense vegetation that can obstruct water flow. Fuel reduction is essential in the Everglades, where water movement is critical to the health of the entire ecosystem. Historically, natural fires were a common occurrence in the Everglades.

**Fig. 1:**
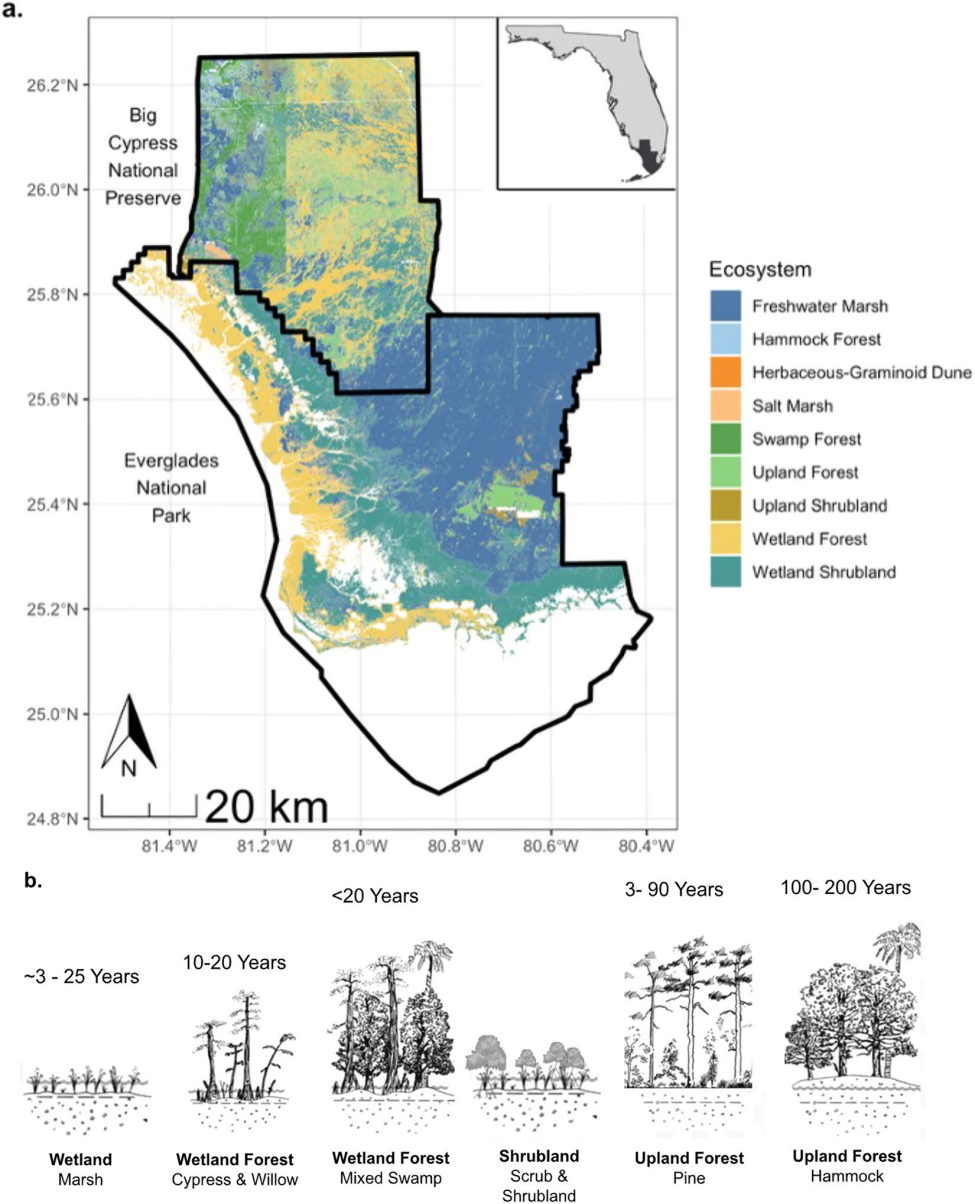
Everglades ecosystems (**a**) and the historical range of fire return intervals found for major ecosystems in the southeastern U.S.^17,45–48,57^ (**b**). The vegetation map (**a**) is from the Vegetation Mapping Inventory Project of Everglades National Park and Big Cypress National Preserve^58–61^.

Across the Everglades landscape, fire, climate variability^39^, and hydrology^25^ are closely linked, playing a crucial role in ecosystem heterogeneity through complex feedback loops^27,40,41^. Fire influences the distribution and composition of Everglades plant communities^16,17,42,43^, and their historical fire return intervals are characteristic of ecosystem structure and biomass (Fig. 1). Short-stature wetlands, freshwater marshes, and wet prairies burn every 3 to 25 years and can recover from fire within a few years. Wetland forests experience a wide range in fire return intervals. Although mangroves are not generally influenced by fire^16,44^, cypress (*Taxodium distichum*) and mixed hardwood swamps historically burned every 10–20 years^16,45^. Fire-maintained wetland forests include cypress domes, cypress prairies, and dwarf cypress savannas. The historical fire return interval for upland forests of south Florida slash pine (*Pinus elliottit* var. *densa*) is ~3–90 years^17,46–48^. Pinelands depend on frequent, low-intensity surface fires to maintain their species composition and structure^18^. Endemic pineland herbs and grasses can be shaded out within 5 to 10 years without fire by encroaching hardwood vegetation, supporting the eventual transition to hammocks. Upland hardwood forests, known as hammocks, are much less extensive and are typically found where there is an accumulation of organic matter on elevated limestone platforms^49^. Fire rarely penetrates hammocks beyond their edges except under severe wildfire conditions, with core areas thought to have historically burned as infrequently as every 100–200 years^16,17^. Hammocks and pine forests occupy opposite ends of a fire-adaptation spectrum.

Here, we define the contemporary fire regimes of the sub-tropical ecosystems of the Florida Everglades using patterns in both wildfires and prescribed fires over the last + 40 years. We identify fire patterns and trends that can signal changes in essential ecosystem structure and function across the Everglades landscape. We provide fire history layers for Everglades National Park (ENP) and Big Cypress National Preserve (BCNP) to support planning and restoration evaluation.

## Methods

### Study area

The Florida Everglades experience a year-round growing season, characterized by distinct wet and dry seasons. Although variable, 60–80% of annual precipitation (130–160 cm) occurs between May and October^16,27,50^. The mean annual temperature in this region is 24 °C, ranging from 17 °C in January to 40 °C in August. The Everglades landscape consists of a mosaic of plant communities, the distribution of which is dictated by subtle changes in elevation, hydrology, and fire history^18^. Everglades National Park and Big Cypress National Preserve (hereafter referred to as the Everglades) account for a total area of 9,057 km^2^.

### Fire perimeters

Since the establishment of Everglades National Park and Big Cypress National Preserve, fires have been recorded. Although the information within fire report forms changed over time^23^, Wildland Fire Report (WFR) forms were used to develop a database in 2003^23^. These databases were created using fire reports that go back to 1948 for ENP and 1978 for BCNP. Information in the fire record databases was based on the Proposed National Park Service Fire GIS Data Standards, as provided by the Southeast Regional Fire GIS Specialist, and predates the National Wildfire Coordinating Group data standards^51–53^. Spatial information about the fires was derived from various sources, including hand-drawn maps in the paper fire records, hand-drawn mylar maps, and digital data layers^23^.

In the development of the fire database, fire perimeter polygons were constructed using the spatial information associated with Fire Report Forms^23^. When digital perimeters were available, they were compared to the paper maps associated with the fire reports and checked for accuracy. When a fire did not have a map demarcating a perimeter, coordinate information and standard shapes were used as proxies for the fire perimeter, based on a strict set of criteria. Circles were used when the burned acreage estimate from the fire reporting form was less than 0.4 hectares. Rectangles were used for estimated burned acreages larger than 0.4 hectares and were sized to equal the acreage reported. These circular and rectangular fire “perimeters” were positioned geographically based on information obtained from the fire narrative. Fire perimeters from 1994 to 2004 were visually compared against Landsat archive satellite imagery as part of an evaluation of the use of satellite data to map Everglades fire history^23^. Cloud-free satellite images following the occurrence of each fire were inspected to evaluate fire scars that were large enough to be visible in Landsat imagery (fires > 1.2 hectares). A small percentage of polygons were misplaced, verifying that the polygons in the reporting form were accurate. When errors were found, polygons were corrected in the database^23^. Fires for which no scar was apparent in the imagery were also documented. All locational errors were noted in the database.

The database was organized into ESRI ArcGIS (Environmental Systems Research Institute, Inc., Redlands, CA) feature datasets. Each feature dataset included a polygon feature class that included a comprehensive table of information about the fire. We obtained fire perimeters formatted as ESRI shapefiles that were compiled into annual fire perimeter files from ENP and BCNP. The data in these files included the spatial extent of fires, fire type (wild or prescribed), the date of discovery or ignition, and the date of control or completion. We standardized and harmonized the data, as the column names and information recorded in the files have changed over time and differed for the ENP and BCNP. All data harmonization was done using the *sf* package in R^54^. In this evaluation of fire history, we used information from 1978 to 2023 for both ENP and BCNP^55^. As digital fire perimeter mapping was shown to be accurate compared to Landsat fire scars^23^, no additional validation was done on the Everglades fire perimeters.

### Contemporary fire history

Contemporary fire history was quantified by evaluating fire patterns at a 30-meter resolution (Table 1). We summarized the total area burned by wildfires and prescribed fires. Rasterizing annual fire perimeters to a 30-meter grid aligned with Landsat, we determined the number of fires that have occurred since 1978 and the number of years since the last fire by creating annual gridded layers using the t*erra* package. We validated fire history layers using 2,500 random points, where we extracted fire frequency information directly from fire perimeters and compared it to the fire frequency recorded at a 30-meter gridded resolution. We compared the observed (fire perimeter-based) and predicted (gridded 30-meter fire frequency), reporting the coefficient of determination and the p-value.

Trends in burned area and fire frequency were determined by measuring changes over time using ordinary least squares (OLS) and non-parametric approaches^56^. For the OLS, we used the lm function to perform a linear regression. The slope coefficient from the regression model was used to indicate the rate of change over time, capturing the trend as a linear slope value. The sign of the slope indicated the trend direction, and the slope value denoted the strength of the trend.

We explored three non-parametric approaches to trend evaluation: Spearman’s rho, Mann–Kendall, and Sen’s slope. The Spearman’s rho is a coefficient of rank correlation used to examine whether an association exists between two variables^56^. Under the null hypothesis, there is no trend. If the estimate is positive, we conclude that there is an increasing trend; if negative, we conclude that there is a decreasing trend. The Mann–Kendall approach is insensitive to the existence of seasonality^56^. A positive estimate is indicative of a positive trend, while a negative value indicates a negative trend. With Sen’s slope, the sign of the slope estimate shows the trend direction, and the slope value indicates the strength of the trend. All analyses were done in R^54^. While we provide all estimates of trend to show consistency in the results regardless of the approach used, we use Sen’s slope when discussing the magnitude of trend in the results.

**Table 1:** Fire history layers generated from the fire perimeter files of Everglades National Park and Big Cypress National Preserve (1978–2023)^55^.

Fire History Variable	Description
Total Area Burned	The annual area burned (km^2^)
Total Fires	The number of fires since 1978
Time Since Fire	The number of years since the last fire (1978–2023)

## Data Records

The Everglades fire perimeter and history dataset consists of harmonized fire perimeter shapefiles and two GeoTIFF raster files that contain the spatial extent of fires in Everglades National Park and Big Cypress National Preserve from 1978 to 2023^55^. The fire perimeter shapefile comprises 5,394 records and includes information on the National Park file from which the data originated, the fire unique identifier, fire number, fire name, year, date of discovery, date of control, flag for missing date, type of fire (prescribed or wildfire), and spatial extent.

The fire perimeter shapefiles were rasterized to 30-meter Landsat grids to create two GeoTIFF raster files that show the burned area annually. One GeoTIFF file contained 46 layers, which represent the annual burned area from 1978 to 2023, where the burned area was denoted by cells with a value of 1. This file is useful for calculating frequency-related fire history metrics. The second GeoTIFF was similar, except the burned area was denoted by cells with the same value as the year the fire occurred. These fires are essential for calculating time-based metrics, such as time since the fire. These three datasets have been archived at the Environmental Data Initiative (EDI) data repository^55^.

## Technical Validation

**Fig. 2:**
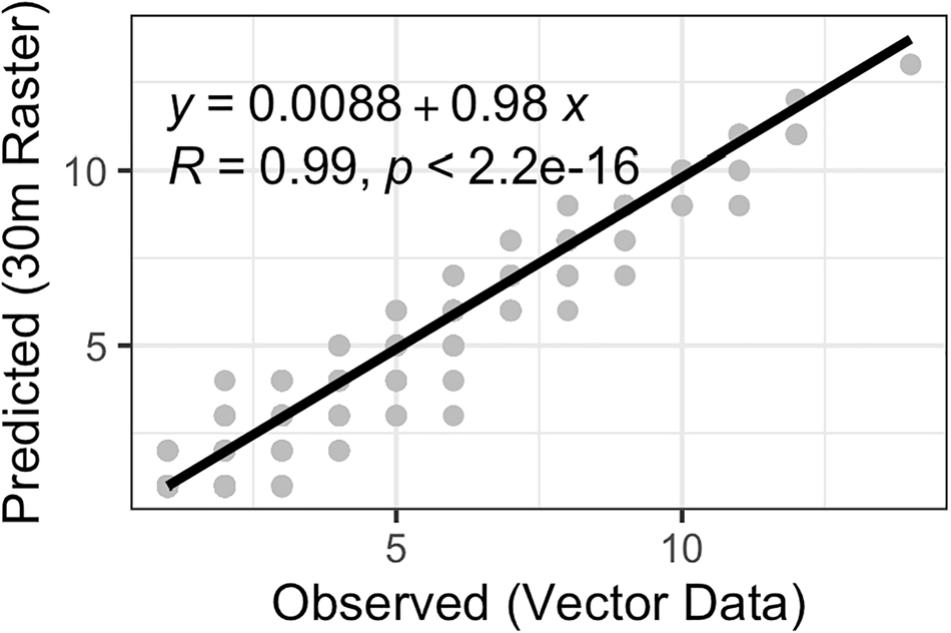
Observed versus predicted total fires for 2500 validation points distributed across the Everglades. The observed data were derived from the fire-perimeter vectors, and the predicted data were extracted from the gridded 30-meter fire frequency layer^55^.

To understand how well fire history can be represented at a 30-meter resolution, we compared observed and predicted total fires at 2500 randomly sampled locations across the Everglades (Fig. 2). Observed total fires from 1978 to 2023 ranged between 1 and 14, and total fires from the 30-meter fire history layers ranged from 1 to 14, with a mean difference of 0.5 fires. The gridded 30-meter fire history layers were found to be representative of the vector-based data. The R² for observed total fires versus predicted values was 0.99 (p < 0.001), providing confidence that generating fire history layers at a 30-meter resolution was sufficient to maintain spatial and temporal variation in fire history across the Everglades.

**Fig. 3:**
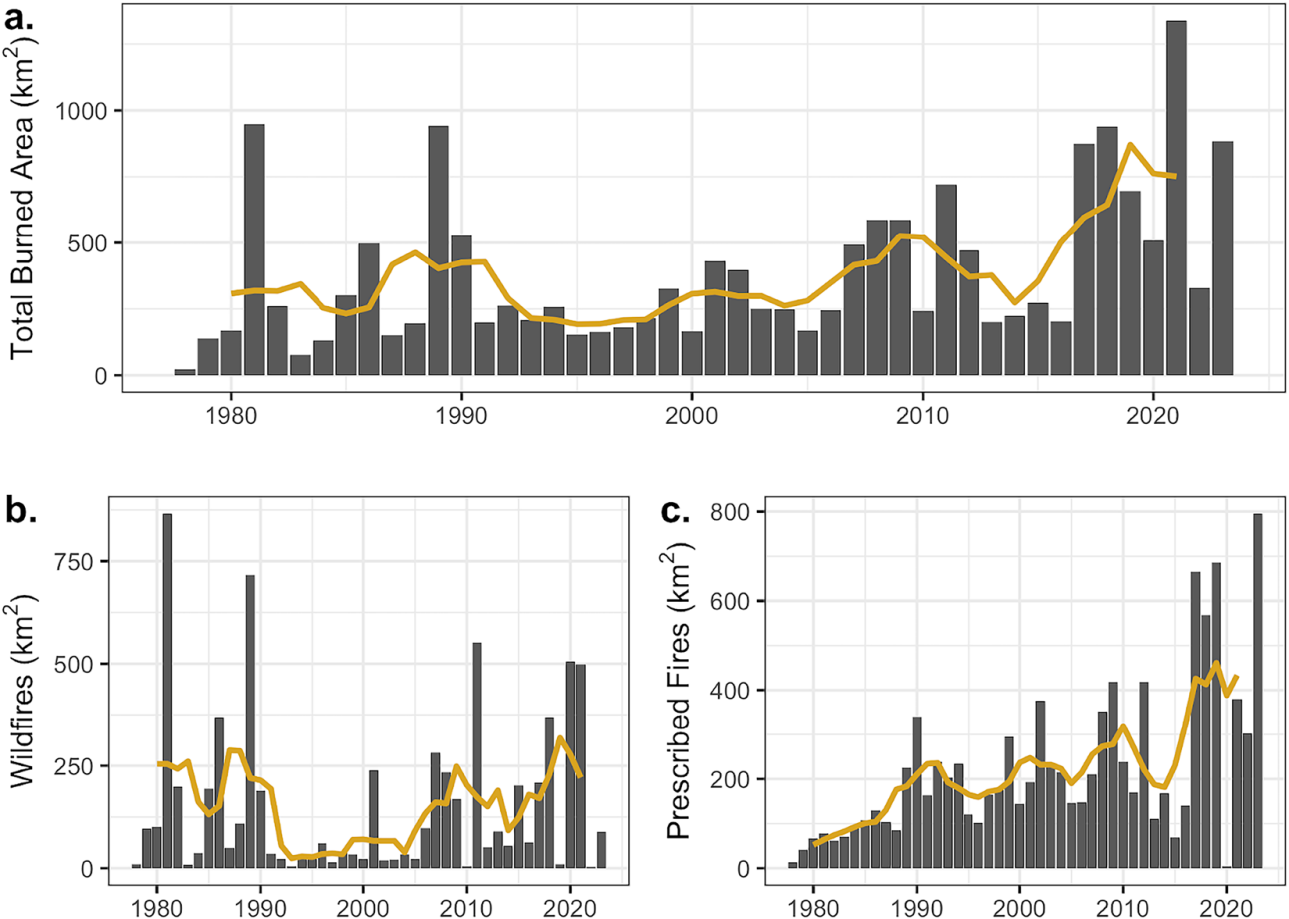
Annual total burned area (km^2^; **a**) for wildfires (**b**) and for prescribed fires (**c**) across the Everglades. The 5-year rolling mean is shown as a yellow line.

The total annual burned area was highly variable across the Everglades, with a strong increasing trend evident in the 5-year rolling mean (Fig. 3). Trend detection using different methods yielded consistent patterns across measures of fire history (Table 2). Annual burned area ranged from 22 to 1,338 km^2^, and the trend from 1978 to 2023 was 7.3 km^2^ Yr^−1^ (Table 2). Both wildfires and prescribed fires made significant contributions to the total burned area. The mean contribution of wildfires was 40% over the entire study period. While there was no increasing trend in wildfires, a strong increasing trend was observed in prescribed fires, covering 6.35 km^2^ Yr^−1^(Table 2).

**Table 2:** Estimates of the trend in fire across the Everglades landscape using ordinary least squares (OLS), Spearman’s rho, Mann–Kendall, and Sen’s slope.

	OLS		Spearman’s rho		Mann–Kendall		Sen’s Slope	
	Estimate	Pvalue	Estimate	Pvalue	Estimate	Pvalue	Estimate	Pvalue
Total Burned Area	9.341	0.003	0.492	0.001	0.372	p < 0.001	7.268	p < 0.001
Burned Area (Wildfires)	0.337	0.88	0.076	0.613	0.053	0.611	0.609	0.609
Burned Area (Prescribed Fires)	7.792	p < 0.001	0.58	p < 0.001	0.455	p < 0.001	6.35	p < 0.001
Fire Size	0.223	p < 0.001	0.724	p < 0.001	0.561	p < 0.001	0.135	p < 0.001
Area: Perimeter	3.418	p < 0.001	0.65	p < 0.001	0.484	p < 0.001	2.822	p < 0.001

**Fig. 4:**
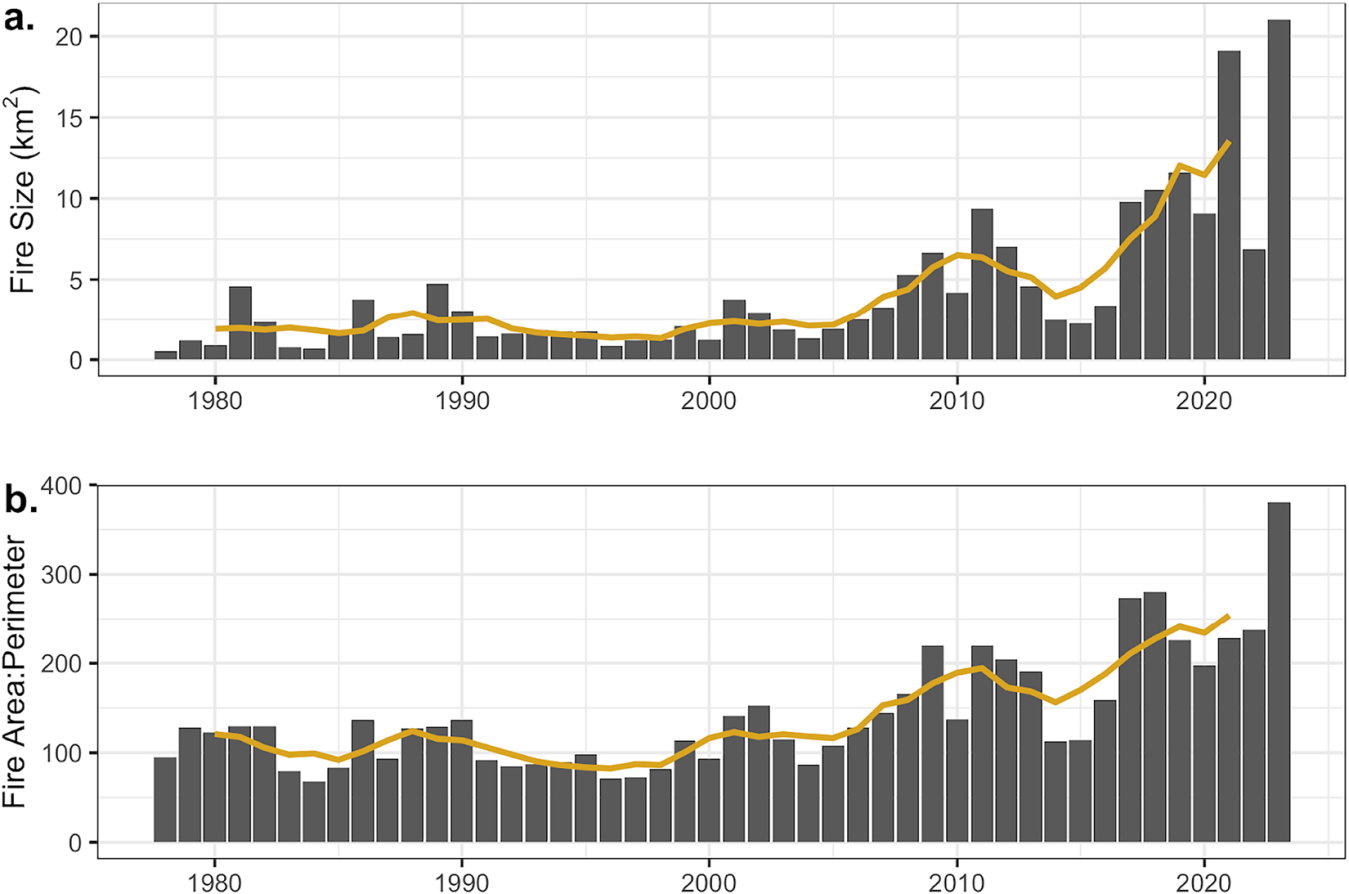
Fire size (km^2^; **a**) and the ratio of fire area to perimeter (km; **b**) for prescribed and wildfires across the Everglades. The 5-year rolling mean is shown as a yellow line.

The size of fires increased over time in the Everglades region (Fig. 4), with a trend of 0.135 km^2^ Yr^−1^ (Table 2). The mean fire size was less than 2 ± 0.2 km^2^ before 2001 and increased to 6.6 ± 1.1 km^2^ from 2010 to 2023. Similar to the mean fire size, the fire area to perimeter ratio also had a strong positive trend of 2.82 km Yr^−1^ (Table 2; Fig. 4). Considering the increasing role of prescribed fires (Fig. 3), the footprint of land management decisions through prescribed fire has influenced patterns in contemporary fire regimes.

**Fig. 5:**
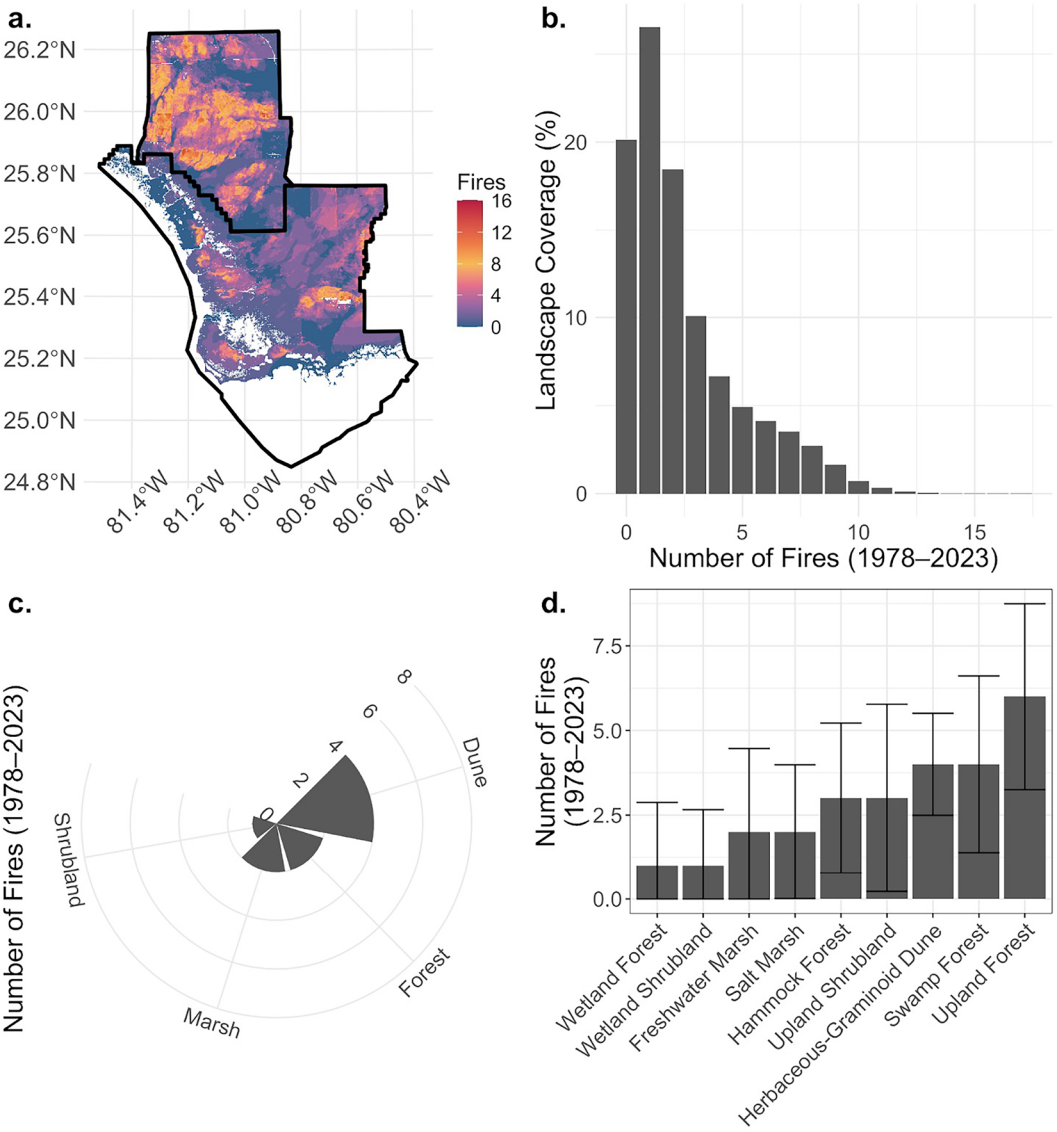
Total number of fires (**a**) by the percent coverage (**b**), vegetation types (**c**), and by ecosystem structure categories and classes (**d**) across the Everglades landscape.

**Fig. 6:**
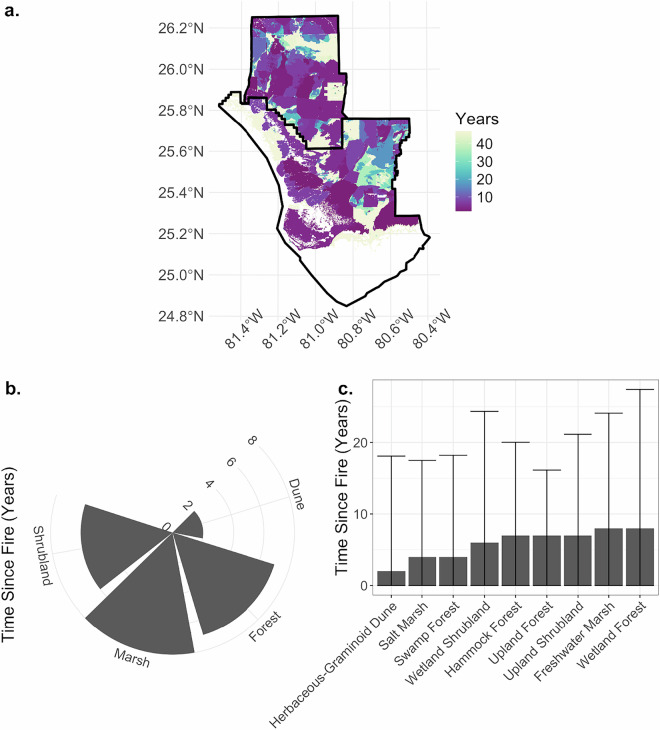
The number of years since the last fire across the Everglades landscape (**a**), for ecosystem structure categories (**b**), and vegetation types (**c**).

Locations across the Everglades have experienced variations in fire activity since 1978 (Fig. 5). The total number of fires for a location ranged from 0 to 17. While 20% of locations had not burned during the study period, most of the landscape experienced at least one fire. The median number of fires was greatest for upland forests and lowest for wetland forests. Although dunes represented a very small component of the Everglades landscape, they had the highest median number of fires, followed by forest and marsh. The years since the last fire ranged from 2 to 47 (Fig. 6). Across ecosystems, the median number of years since fire ranged from 2 to 8. Dunes had the lowest time since fire, followed by marsh and swamp forest. The median number of years since the fire was 7 years for upland ecosystems.

## Usage Notes

Resource manager prescriptions have become increasingly important in this region for maintaining the health and resilience of fire-adapted ecosystems^25,35^. Support for these activities could have a substantial impact on the structure and potentially the function of the Everglades landscape^25^. Contemporary fire regimes of the Everglades consist of similar proportions of wildfires and prescribed fires. Historically, most lightning-ignited wildfires occur during the transition from the wet to the dry season (April to June), when fuel moisture is low enough to promote fire spread and there is an increase in lightning from convective storms. As the climate continues to change, these patterns are shifting. Currently, prescribed fires implemented by the National Park Service are performed throughout the year to balance the need to maintain natural patterns^6^ with requirements for using fire under conditions where risks are minimized. This study has developed resources to map fire dynamics in space, evaluating patterns in ecosystem resilience and restoration success, and highlighting regions where fire patterns do not align with vegetation types.

## Data Availability

The code is available on GitHub: https://github.com/Malone-Disturbance-Ecology-Lab/Everglades-Fire-History.
